# Community perceptions of malaria and vaccines in two districts of Mozambique

**DOI:** 10.1186/1475-2875-11-394

**Published:** 2012-11-28

**Authors:** Allison Bingham, Felisbela Gaspar, Kathryn Lancaster, Juliana Conjera, Yvette Collymore, Antoinette Ba-Nguz

**Affiliations:** 1 PATH Kenya, PO Box 19128–40123, Mega City Building, Mezzanine Floor, Along Nairobi Road, Kisumu, Kenya; 2 Traditional Medicine Institute, Ministry of Health - Av: Eduardo Mondlane/Salvador Allende, PO Box 264, Maputo, Mozambique; 3 Department of Epidemiology, UNC-CH, Campus Box 7435, Chapel Hill, NC, 27599-7435, USA; 4 M&E, FHI360, Rua dos Sinais No 50/74, Maputo, Mozambique; 5 PATH Malaria Vaccine Initiative (MVI), 455 Massachusetts Ave., Washington, DC, NW, 20001-2621, USA; 6 PATH Malaria Vaccine Initiative, ACS Plaza, 4th floor, Lenana and Galana Road, PO Box 76634, Nairobi, 00508, Kenya

**Keywords:** Formative studies, Vaccination, Malaria, Children, Communities, Health communications

## Abstract

**Background:**

Malaria is a leading cause of mortality and morbidity in Mozambique, with nearly three-quarters of the country’s malaria-related deaths occurring in children younger than five years. A malaria vaccine is not yet available, but planning is underway for a possible introduction, as soon as one becomes available. In an effort to inform the planning process, this study explored sociocultural and health communications issues among individuals at the community level who are both responsible for decisions about vaccine use and who are likely to influence decisions about vaccine use.

**Methods:**

Researchers conducted a qualitative study in two malaria-endemic districts in southern Mozambique. Using criterion-based sampling, they conducted 23 focus group discussions and 26 in-depth interviews. Implementation was guided by the engagement of community stakeholders.

**Results:**

Community members recognize that malaria contributes to high death rates and affects the workforce, school attendance, and the economy. Vaccines are seen as a means to reduce the threat of childhood illnesses and to keep children and the rest of the community healthy. Perceived constraints to accessing vaccine services include long queues, staff shortages, and a lack of resources at health care facilities. Local leaders play a significant role in motivating caregivers to have their children vaccinated. Participants generally felt that a vaccine could help to prevent malaria, although some voiced concern that the focus was only on young children and not on older children, pregnant women, and the elderly. Probed on their understanding of vaccine efficacy, participants voiced various views, including the perception that while some vaccines did not fully prevent disease they still had important benefits. Overall, it would be essential for local leaders to be involved in the design of specific messages for a future malaria vaccine communications strategy, and for those messages to be translated into local languages.

**Conclusions:**

Acceptance of routine childhood vaccines bodes well for a future malaria vaccine. Vaccinating children is a well-established routine that is viewed favourably in Mozambique. A communications strategy would need to build on existing immunization efforts and use trusted sources—including current government dissemination arrangements—to deliver health information.

## Background

Nearly 216 million people suffered from episodes of malaria worldwide in 2010, and an estimated 655,000 people died because of the disease. An estimated 86% of the deaths were among children younger than five years, with the overwhelming majority occurring in sub-Saharan Africa [1]. In 2011, Mozambique had an estimated 3.3 million malaria cases [2]. Of these, an estimated 20,490 people died, with 15,000 of those deaths occurring among children younger than five. Malaria parasite transmission continues throughout the year, with the highest rates at the end of the rainy season from December to April [2]. The country’s temperature and rain patterns, abundant mosquito breeding sites, and poor housing are primary contributing factors [3]. As in other parts of Africa, malaria often affects children’s mental and physical development and is a leading cause of school absenteeism. The disease can have a debilitating effect on adults as well, often removing them from the workforce for days or weeks at a time [1].

Mozambique has a multi-pronged approach to addressing malaria, including the use of insecticide-treated nets (ITNs), indoor residual spraying with insecticides, and intermittent preventive treatment for pregnant women. The Roll Back Malaria Initiative for the African Region was introduced in 1998, with strategies that promote civil society involvement in health and that build on the family’s capacity to prevent, recognize, and manage malaria appropriately. This strategy targets children younger than five and pregnant women. Some prevention initiatives are showing progress. For example, the country is closing in on the goal of 95% ITN use among pregnant women. ITN use within this group increased to 92% in 2011, compared with roughly 77% in 2009 and 2010 [2].

No vaccine against malaria currently exists, but recent advances in malaria vaccine development have heightened the possibility that, if proven efficacious, such a vaccine could be deployed for use with existing interventions. The malaria vaccine community is working toward a 2015 milestone of licensing a first-generation vaccine [4], and a number of African countries are already determining the kinds of information they would need to make timely and evidence-based decisions on vaccine introduction, as soon as the first one is available [5]. Regarding the use of vaccines for other diseases, Mozambique’s Expanded Programme on Immunization, initiated in 1979, focuses on children less than one year of age and on pregnant women. Services are offered at approximately 870 health centres (representing 86% of health units in the existing primary and secondary network of fixed vaccination sites); however, only about one-half of the country’s population is served by the existing health network. Many health service programmes lack trained available staff and adequate transportation for outreach events [6]. To expand the reach, the country uses monthly health days and the World Health Organization (WHO) Reach Every District strategy. In 2011, 73% of one-year-olds were fully immunized, compared with 63% in 2003 [2].

In light of progress on malaria vaccine development, Family Health International (FHI) worked with the PATH Malaria Vaccine Initiative in collaboration with Mozambique’s Ministry of Health and the Institute of Traditional Medicine to explore sociocultural and health communications issues in an effort to understand the factors that could affect future malaria vaccine acceptance. The WHO framework for new vaccine introduction calls formative research a key step toward vaccine introduction [7]. Formative research provides a solid evidence base for, among other things, designing an effective communications strategy.

It is widely accepted that a well-planned communications strategy, informed by formative research, can facilitate achievement of vaccine coverage objectives and build trust for vaccine acceptance [7-11]. Information delivered through appropriate channels and trusted sources can generate support for and acceptance of vaccine programmes among local decision-makers and help immunization programmes maintain coverage targets. Effective communication, especially in hard-to-reach populations, is essential for building acceptance of vaccines among groups who may question them. If left unaddressed, concerns about a vaccine expressed by parents and other key influencers could foster negative attitudes toward immunization and inhibit vaccine acceptance. 

This study aimed to gather information that would inform the design of a communications strategy, including ways to engage communities well before a malaria vaccine becomes available, and to provide policymakers with data from target communities to guide efforts related to possible vaccine introduction. Specifically, the study addressed the following questions:

1. What key perceptions, beliefs, attitudes, and health practices are held in relation to malaria and vaccines at the community level in Mozambique, and what information gaps exist?

2. What are the primary household decision-making processes and treatment-seeking patterns related to malaria and vaccination?

3. Who are the key audiences related to child immunization in communities?

4. What are the primary communications and information channels (related to child health) at the community level?

## Methods

### Site selection

Using criterion-based sampling, researchers selected two districts in Mozambique that met the following criteria: (1) neither district should be hosting an ongoing malaria vaccine clinical trial; (2) both should be as accessible as possible from Maputo during the rainy season when the study would take place; (3) the selection should allow for geographic and cultural diversity; and (4) the sites should exhibit high malaria endemicity. Taking these and other criteria, including cost implications, into account, it was decided to limit the sites to the southern part of the country but to identify one district on the coast and one inland. Differences in geography and culture exist between the coastal and inland regions of Mozambique, even though the country’s extreme north–south elongation makes it difficult to capture all of the cultural and subcultural variations in a small study.

Researchers selected Chókwè District in Gaza Province and Massinga District in Inhambane Province. Massinga is on the coast; Chókwè is inland, with a large irrigation canal running from the Limpopo River in the south to Chilembene community in the southeast. The two districts are home to highly mobile workers who travel out of Mozambique for work in South African mines. Labour migration is usually associated with shifts in responsibility for both household decision-making and household income. The migrant workers usually remit funds to relatives back home [12]. Migration into Mozambique is also a feature of the region, contributing to the country’s enormous ethnic and linguistic diversity [13].

Facilities with child health clinics were identified for the study, and two were selected for each district—one serving a semi-urban population in the district capital and the other serving a rural population. If more than one rural or urban clinic met the selection criteria, researchers used a table of random numbers to select the clinic. Similarly, one semi-urban neighbourhood or *bairro* was randomly selected from each district and a block or *quarteirão* selected from each *bairro.*

### Participant selection and data collection

This formative study was conducted among individuals at the community level who were felt to be either responsible for individual decisions about childhood vaccine use or who were felt to have the potential to influence those decisions. Participant selection was largely purposive (criterion based). Selection was guided by a social ecological conceptual framework [8] that recognizes categories of people that can influence whether a child is immunized. These categories constitute important target audiences for developing a health communications strategy aimed at engaging communities in new vaccine introduction activities. They include the “direct” decision-makers of childhood vaccine use (parents and other caregivers) as well as those who may have an important influence on them (health providers, religious leaders, traditional healers, traditional birth attendants, and leaders of nongovernmental organizations [NGOs]).

Focus group discussion (FGD) and in-depth interview (IDI) techniques were used to collect data between May and June of 2010. Researchers completed a total of 23 FGDs (including 250 participants) and 24 IDIs (see Table 1). FGDs were used to explore the knowledge and beliefs of both “direct” decision-makers and those with the potential to influence decisions. Interviews were conducted with those who provide services and conduct malaria prevention programmes, such as heads of local service units and NGOs.

**Table 1:** Sample framework and completed data collection events

**Total N participants = 200**	***Chókwè***	***Massinga***	***Total events***
**Total focus group discussions**			**23**
Female caregivers, rural	1	1	2
Female caregivers, urban	1	1	2
Male caregivers, rural	1	1	2
Male caregivers, urban	1	1	2
Other caregivers, rural	1	1	2
Other caregivers, urban	1	1	2
Traditional healers	2	2	4
Traditional leaders	1	2	3
Religious leaders	2	2	4
**Total in-depth interviews**			**26**
Head of immunization	1	1	2
Community health worker	1	1	2
Director of health unit that provides preventive services	2	2	4
Member of the Association of Traditional Medics of Mozambique	1	1	2
Key informant at community level (Community block/area leaders)	4	4	8
Traditional birth attendant	2	2	4
Leader of nongovernmental organization	2	2	4
**Total data collection events**	**24**	**25**	**49**

The local research team consisted of the local principal investigator, the research coordinator, the field coordinator, one transcriptionist, and two interviewers. Interviewers were fluent in both Portuguese and one of the two local languages spoken in the study districts (Xichangana in Chókwè and Xitshwa in Massinga). In some instances during data collection events, the interviewers simultaneously translated the questions from Portuguese to the local languages.

Researchers carried out thematic content analysis using a team-based iterative approach to qualitative research developed by FHI researchers. An analyst developed structural content codes based on interview guides that had been developed and fine-tuned during training. A draft codebook was then developed, reviewed by the local research team and PATH researchers, and then finalized. FGDs and IDIs were recorded, transcribed, and translated from the local languages to Portuguese and then to English. Using the updated codebook, all of the English translated transcripts were coded in the software program NVivo by two trained analysts from FHI. Throughout the coding process, the local principal investigator and field coordinator, both fluent in English, Portuguese, Xichangana, and Xitshwa, reviewed original local-language transcripts against English code reports to verify codes, identify and discuss discrepancies in coding, findings, and interpretation. During manuscript preparation, all statements were located in the transcripts and reviewed to ensure their meaning was interpreted correctly. In addition to reviewing code reports for “themes,” the analyst specifically looked at report information by subgroup (such as caregivers and service providers) and by site, gender, and religious and ethnic group.

Ethical approval was received from three institutions for this study: FHI’s Protection of Human Subjects Committee, the PATH Research Ethics Committee, and the Mozambique Ministry of Health’s National Bio-Ethics Committee.

As part of the consenting process, researchers described the impact of malaria globally, noting that scientists were testing malaria vaccines to see if they could offer another method to be used with current strategies for controlling the disease. Community memebers heard that their views and experiences on malaria and vaccines would help national leaders plan for decisions on a possible malaria vaccine.

## Results

Overall, the findings suggest that communities see malaria as part of everyday life and are familiar with most of the disease’s symptoms. While they voiced confidence in the country’s childhood immunization programme and generally viewed vaccines in a positive light, they cited the need for more information about particular vaccines and raised specific concerns about vaccine services. In addition, they raised many expectations and questions about a possible malaria vaccine.

Findings from the FGDs and IDIs are presented below according to the key domains of inquiry which guided this study (see Table 2). Observable differences among groups and between districts are highlighted where they exist.

**Table 2:** Key areas of inquiry covered in the study

**Theme**	**Key questions**
Perceptions and experiences with malaria	· What is the impact of malaria on the community?
	· What are the local terms for malaria?
	· What are the symptoms of malaria?
	· What are the ways to prevent and treat malaria?
	· What malaria programmes are available?
	· How well do they work?
Perceptions and experiences with vaccination	· What is the understanding of vaccination?
	· What are the benefits of vaccines?
	· What influences the decision to vaccinate children?
	· What prevents the decision to vaccinate children?
	· Who might discourage vaccinations?
Health decision-making in households	· Who are the primary decision-makers for childhood health, including vaccination?
	· Who makes the decision if the primary decision-maker is not present?
Acceptability of a future malaria vaccine	· What concerns exist?
	· What information is needed?
Trusted sources and information channels	· Where do you get your health information?
	· Who are the trusted sources within the community?

### Perceptions and experiences with malaria

#### Malaria’s impact on the community

Study participants voiced the view that malaria had a negative impact on the community. Nearly all the participants referred to high death rates related to malaria and to the overall loss of human resources in the workforce and in the fields or *machambas*. Additionally, many noted that malaria severely affected attendance at school and work and the production of crops.

*“Most of the time, people will miss work or other such activities, as they are made to stay home. They are unable to go to work or the field, thus the production of the crops suffers.”* (Head of community health worker programme from Chókwè)

#### Malaria terminology

The most commonly used terms for malaria in both Chókwè and Massinga refer to either mosquito or fever. For example, *dzedzedzé*, *dsedse*, *mudinhane*, *muzototó*, and *ximungwamugwane* all refer to mosquito. The term *efevere* references fever. The term ‘malaria’ is also used in Portuguese.

#### Malaria signs, symptoms, and treatment-seeking behaviour

When asked what symptoms they associated with malaria, community members and caregivers most commonly reported fever, chills, vomiting, loss of appetite, and weakness, and several also mentioned diarrhoea and coughing. Caregivers often noted that children who fell sick with malaria would have warm bodies and would want to stay in the sun or under a blanket. Parents may even describe the child as being ‘lazy.’ One woman mentioned that when her child refused to breastfeed, she knew the child had malaria. A few also noted that malaria-infected individuals became delirious—“saying things that no one can understand when malaria reaches the head.” In listing the symptoms, a male caregiver referred to “many kinds of malaria.”

*“There are many kinds of malaria. Some people when they get malaria, their body becomes weak, their joints paining, vomiting, feel cold, and sometimes they feel hot. They cover with the blanket. So these are different kinds of malaria.”* (Male caregiver from Massinga)

While a few focus group participants said they associated convulsions in children with malaria, others said they did not. Participants explained that convulsions—which some described as collapsing and fainting—are seen as a manifestation of a local illness that is associated with the presence of a full moon. This illness is referred to locally as *nweti*, *nhocani*, or *tynhocani.* Participants said that caregivers may not take the child to a clinic or hospital immediately. They may prefer instead to seek traditional healers or home remedies. Caregivers reported that they would seek hospital treatment for convulsions if treatment from traditional healers failed.

*“We know that it is “nweti” (moon sickness), because when the sickness starts, the child begins to suffer from constant collapses and is always fainting. We usually treat “nweti” by preparing a medicine made from leaves and then we take the child to the hospital.”* (Male caregiver from Chókwè)

The majority of community members and caregivers reported that their first response to the appearance of malaria-like symptoms was to take the sick child to the hospital for diagnosis and treatment. Participants generally spoke of taking action when a child’s body “over-heats” or needs to be “cooled down.” At the same time, many caregivers said they may first try to manage malaria symptoms at home. The most common treatment was the over-the-counter medicine paracetamol and the use of cold water or wet cloths “to cool off the body.” When these efforts failed, medical treatment at a hospital was sought. Traditional remedies were also discussed within the context of treatment for malaria, although participants noted that they are less commonly used today. Such remedies included the drinking of boiled herbs or plant leaves, including avocado and eucalyptus leaves.

Health staff and religious leaders recognized the impact of delays in treatment for convulsions, fever, and other malaria-like symptoms. The findings show that such delays are likely because some symptoms may not be associated with malaria. Underlying many comments was the fear that if the child with convulsions was taken to the hospital and given an injection, the child would die. One health centre director noted:

“They say that children with convulsions should never be taken to the health centre. If they are taken, they will be given injections and if given the injection, the child will die…”

Another health provider recalled that in some areas in Chókwè when a child has convulsions, families prefer to take the child to the ash pile (where household cooking fire ash is thrown out) and will leave the child there until the convulsions stop. The belief also follows that contact with the child’s urine or faeces could result in transmission of the convulsions to another person.

One traditional healer stressed the view that while hospitals are meant to treat certain kinds of illnesses, traditional healers were better positioned to treat perceived spiritual illnesses or other ailments long known to the community. The healer referred to moon sickness as the kind of ailment treated using medicinal plants and traditional ceremonies.

*“Some people believe that convulsion is the disease of the full moon, such that during a certain time of the month, if the child does not complete his traditional treatment, it will lead to convulsion. When this happens, the parents have to seek help from an experienced traditional healer who can cure it. Here the child has to complete the treatment. Malaria is not in any way related to or causing convulsions in a child.”* (NGO leader from Chókwè)

#### Malaria prevention practices and views on transmission

Caregivers and other community members said that the main household prevention methods for malaria included the use of mosquito nets and the cleaning of living areas. They spoke of cleaning up trash, preventing the build-up of stagnant water, trimming trees and grass around the house, and burning herbs and leaves to repel mosquitoes. Researchers observed that household compounds were generally free of debris and garbage.

Most of the prevention methods mentioned suggested a basic understanding that mosquitoes transmit malaria, with some comments linking transmission to the condition of living areas. For example, a male caregiver from Massinga said that the disease was caused by mosquitoes and “rubbish.” Other comments linked transmission to personal hygiene.

Community members also described prevention programmes organized by the government and community. Participants from Chókwè spoke of the door-to-door spraying of homes—known as “pulverization.” Organized door-to-door spraying, which is not undertaken in Massinga, is a principal component of the government-organized malaria prevention programme in Chókwè, and it is conducted roughly every six months. Nearly all the Chókwè participants criticized the spraying programme. Some said they did not believe the spraying worked and revealed that they usually closed their doors and windows to prevent the sprayers from entering their homes. Some participants concluded that the spray is diluted.

*“Pulverization! But the medicine that they apply only give us work after they spray, as mosquitoes continue to enter and bite us, as if they were sent by someone. The pulverization does not work well. They dilute the mixture more with water instead of medicine.”* (Female caregiver from Chókwè)

Community members in both study settings cited campaigns to educate people about household and community methods for malaria prevention. Education sessions were said to be conducted by hospital personnel, with information taught including the use of bed nets and the cleaning of houses. Concerns were also raised about the accessibility of information, education, and communications materials on malaria prevention. These materials, when printed, are accessible only to people who read Portuguese. The local languages used in the settings for this study are both oral and written, and participants cited hymn books as an example of material that is available in the Xichangana and Xitshwa languages.

*“Most information we get is in Portuguese. Most local communities don’t speak Portuguese. It means that the information doesn’t work. It is needed to use a language that people can understand properly. For me, a portion of these materials have to be written in the local language. They have to be disseminated by community leaders, like in a church or in community meetings. That is why I repeat again, we have to train community leaders to guide and disseminate this information either for malaria, TB, or for other health care at community.”* (Health staff member from Chókwè)

### Perceptions and experiences with childhood vaccination

Caregivers and some community leaders in Chókwè and Massinga demonstrated varying levels of knowledge about vaccines that are available in their communities. Roughly half of the respondents—mainly community leaders—named specific vaccines, and they most commonly mentioned measles and tetanus. Among caregivers, some mentioned vaccines that currently do not exist. For example, a few caregivers spoke of malaria vaccines. Overall, most caregivers and members of the community viewed vaccinations as beneficial.

### Motivating factors related to childhood immunization

When asked what influenced them to have a child vaccinated, a consistent theme was the strong desire to keep children and the community healthy and protected against diseases. Most caregivers understood that vaccinations strongly benefitted the child and family. Additionally, many caregivers and religious and traditional leaders indicated that past experiences played a role in their decisions to have a child vaccinated. Some recalled being inoculated as a child and spoke of the perceived benefits of vaccines for measles, tetanus, and polio. There was also a perception that because of vaccines, some diseases no longer represented a threat.

*“The major concern for parents is to have their children vaccinated. They know that the vaccine does well, and even we adults were inoculated when we were children and we didn’t have health problems.” (*Traditional leader from Massinga)

Community members also mentioned adherence to local leadership directives as another influencing factor for childhood vaccinations. Caregivers spoke of the influence of the government, community leaders, and health workers in this regard. Participants stated that vaccination events occurred once volunteers educated the community and encouraged vaccination of children.

“*We the community leaders as soon as we receive information saying that there is a certain work, we are the ones who go out in brigades from block to block where we hold meetings with people and inform them about the vaccine and tell them the days that it will be done so that they can take their children for vaccination. Overall, we expect them to respect the local leadership and adhere to the vaccine.”* (Traditional leader from Massinga)

### Concerns and constraints related to childhood immunization

#### Lack of information or poor messaging

Community members pointed to a lack of information about particular vaccines as one of the most common constraints to having a child vaccinated. They also said they may lack information about vaccination scheduling and times of services. Several participants noted that the information may be available but that it may fail to reach caregivers or that some people may misunderstand what is being communicated. Health care workers and a few caregivers were the ones most likely to highlight the lack of information or unclear messages.

#### Fear of side effects

Closely tied to the perceived lack of information about particular vaccines is the fear of vaccine side effects, or the general fear that an injection or vaccine might harm a child. Community members said they often did not know what discomforts or side effects to expect. Side effects that might discourage caregivers from vaccinating their children ranged from fevers to swelling. Participants often cited side effects that they had observed or had heard about from past campaigns.

*“When a vaccine is wrongly applied, there are children who return abscess and then there is need to explain why the child is abscess because the mother might not go back the other month. There are vaccines that provoke fevers. When the child gets it then becomes feverish, if you do not explain quite well why the child gets feverish, the mother may give up.”* (Head of immunization programme from Massinga)

A traditional healer, reflecting on a past immunization campaign, explained that some parents have associated vaccines with death and the transmission of other diseases:

*“There was a year that some parents refused to take their children to the vaccine because they thought that vaccines were killing their children, that it transmitted other diseases. But lately this belief is already disappearing. When I arrived here, there was a campaign of vaccination. What happened? By speaking here in the neighbourhoods, people used to say that if we take our children for vaccination they will die. But this idea is now disappearing systematically.”* (Traditional healer from Massinga)

#### Taboos related to vaccination

NGO leaders and hospital staff also spoke of lingering taboos in some communities that represent potential constraints to childhood vaccination. One example related to the belief that a newborn should have limited exposure to adults with “hot bodies” or “hot blood” (people who are believed to have recently had sexual intercourse). In this regard, a baby’s illness could be attributed to the child’s exposure to adults with “hot bodies.” Similarly, if a child gets a scar from a vaccine, it may be seen as evidence that the nurse had sexual intercourse the night before. It is believed that a baby should be protected from all “hot-blooded” adults, including parents.

#### Distance to vaccination sites and service hours

A recurring concern among participants was the distance to vaccination services, the long queues when they arrived, and the hours of services. Participants spoke of distance particularly within the context of lacking the money to pay for transportation. Families may also wait in a queue for hours only to be asked to come back the next day. In addition, a family may have to choose between taking valuable time off from farming or other work and having a child immunized. Typically, immunization services are offered at static facilities in the morning, when families work in the fields. Some parents who have tried to do both reported that when they finally arrived at the clinic at 3:00 or 4:00 in the afternoon, services were no longer available. One NGO leader emphasized that health workers were trying hard to make vaccine services more easily accessible for the general community through mobile brigades.

*“Some do not take their children because they go to the farms. They are afraid of losing their production. They prefer going to the farms than taking their children for vaccination.”* (Female caregiver from Chókwè)

#### Quality of service delivery

A few traditional healers, religious leaders, and caregivers spoke of a lack of health supplies at hospitals, unresponsive hospital staff, and poor communication between hospital staff and patients. Religious leaders complained that health workers often were hurried and disrespectful and that they offered little information.

### Understanding the importance of vaccines and how they work

Asked about their general understanding of the purpose of vaccines and how they work, mothers and other participants in FGDs and key informant interviews said they generally saw vaccines as preventing sickness and disease and as helping children to develop and lead healthy lives. Community leaders said they believed that most people had a basic understanding of vaccination being good for disease prevention. Health staff observed that people generally understood that vaccines worked but did not necessarily know the specific diseases that they worked against.

*“When you vaccinate a child to prevent from certain disease she does not get that disease again. She lives well and it takes long for the vaccine to disappear on her body, and when time comes for another vaccination you should take the child and she will live nicely.”* (Male caregiver from Massinga)

In an effort to get to participants’ understanding of vaccine efficacy, particularly partial efficacy, researchers questioned participants on their understanding of how vaccines worked. Responses varied; however, they included the perception that while some vaccines did not fully prevent disease, they still had important benefits. Within this context, several participants, including key informants, traditional leaders, and NGO leaders, spoke of vaccines that worked for a “short time” or “part of the time.” One community leader made the analogy that a vaccine was like gold—even if the protection worked for a short period, it was still “valid.” The word “trust” also surfaced within the context of vaccine efficacy. One key informant noted that people may lose trust in a vaccine if a vaccinated child still contracted the disease.

*“People would not feel good [if vaccinations were only effective part of the time]. But at least for the short time the vaccine exists, they will feel good because the children would grow, they would at least pass the vulnerable age. No, the people would feel good because after a certain period they would apply again.”* (NGO leader from Chókwè)

### Health decision-making in households

Study participants reported that decisions related to vaccinations and other aspects of a child’s health were generally made within the immediate family by one or both parents. Overall, most said that the mother—because of her role as primary caregiver and the person who spent the most time with the children—was primarily responsible for making decisions about a child’s health care. On the other hand, some participants identified the father, because of his role as the head of the household. Other family members who were said to make health-related decisions if the parents were away included grandparents, uncles, aunts, and the eldest child of the family. Researchers noted that community leaders play a critical role in influencing households to have their children vaccinated, especially during campaigns and mobile brigades.

### Future malaria vaccine—expectations

#### Reactions to the possibility of a malaria vaccine

Participants generally expected that a vaccine would help prevent malaria and allow children to lead healthy lives. Caregivers from both Chókwè and Massinga praised the idea and said they would have their children vaccinated to keep them healthy. Several caregivers said they would be eager to receive information about a vaccine as soon as one became available. Service providers and NGOs specifically stated the expectation that a vaccine would reduce malaria-related illnesses and deaths.

#### Questions and concerns about a malaria vaccine

Community members were anxious to know whether, in addition to young children, a malaria vaccine would be introduced for the benefit of adults like them, as well as the elderly and older children. Participants from Massinga voiced the perception that adults—if they are not protected—could pass the disease to their children. Additional fears were expressed that if caregivers became ill, there would be no healthy adults to care for children. Some study participants felt that community members may see a malaria vaccine as a sign that other prevention methods were no longer important. In this regard, a few community leaders were particularly concerned that the community may not continue with existing prevention and control efforts.

*“The disadvantages are related to the effectiveness of the vaccine because at the level of our communities it can be difficult for people to understand why they should take their child for malaria vaccination and yet still use mosquito nets and practice other prevention methods, then to them is like nothing is changed.”* (NGO leader from Chókwè)

Others wanted to know how a future malaria vaccine would work, its duration of efficacy, dosage, potential side effects, who should receive the vaccine, and why (Table 3).

**Table 3:** Information requests

**Information requested by community respondents**	**Supporting quotes**
**Efficacy and dosage**	
· Duration of vaccine efficacy	*“How long does it last when applied to the child?”* Female caregiver from Chókwè
	
**Potential side effects**	*“There is a lot of things that we will like to know before the vaccine comes, so that we feel comfortable taking it. Like what will happen to the child after taking the vaccine?”* Other caregiver from Chókwè
**Target group**	
· Who should be vaccinated	*“Vaccines should also be made for us adults; we want it too.”* Traditional healer from Chókwè
· When will vaccine be available for adults	*“Is the vaccine only for children 0–5 years old or is it for adult as well?”* Director of health unit that provides preventive services
· Will the vaccine be for pregnant women	*“People might ask why the adult {laughs} cannot be vaccinated; this will be a concern for everyone.”* Key informant at community level from Chókwè
Where and how vaccine was tested	*“I think that we the adults should be the first to test this vaccine and only after the results are good we could give the children.”* Traditional leader from Chókwè

### Trusted sources and health information channels

Caregivers, community leaders, and health care providers indicated that reliable processes already exist to inform communities about vaccine services. Participants noted that the government relayed dates for vaccinations to community and block leaders, health care providers, and chiefs, who then made announcements at community gatherings or delivered the information through mobile brigades, by going door to door to reach parents at home with their children, or, as a few participants from Chókwè noted, through a village secretary. Data from this study also indicate that government-sponsored health information channels are fairly consistent and well-recognized. Mass media, specifically television, radio, and mobile units with megaphones, were commonly mentioned as another means of communicating health information. Hospitals also play a central role in announcing new health campaigns. Once the community receives information on vaccination, caregivers take their children—along with their vaccination cards—to the vaccination sites. Many caregivers said the cards, which health officials fill out, provide a means of remembering dates and keeping track of childhood vaccine schedules.

Participants identified trusted and reliable sources of health information as hospital staff, the Ministry of Health, community leaders, *quarteirão* or block leaders, and *bairro* or area leaders. A consistent theme that ran through discussion of this topic was the importance of first informing community leaders—including religious and traditional leaders and the chiefs of ten houses (a local subdivision)—of important vaccine information. These leaders, in turn, relay information to their community in a variety of ways that include going door to door and making announcements at public meeting places. Religious and traditional leaders stressed this vital communication process and voiced their willingness to deliver information.

## Discussion

Key findings in this study should help to inform communications related to a future malaria vaccine. The findings support the view long argued by Nichter and others that any new vaccine would need a communications strategy that builds on acceptance of existing immunization efforts [14]. As seen in the paper by Katahoire *et al.*, an appropriate and relevant health communications strategy can help immunization programmes maintain coverage targets [15]. Such a strategy would, among other things, identify key audiences and appropriate information channels, determine how best to address information gaps, and outline appropriate messages, taking into account potential motivating factors and constraints to immunization.

### Potential motivating factors for childhood vaccination

In general, the evidence points toward broad acceptance of immunization as part of the culture among study participants. The upward trend in vaccine coverage rates reported nationally [2] supports the notion that childhood vaccination is well-established and viewed favourably in Mozambique’s communities. This evidence of broad acceptance is also strengthened by the perception that childhood vaccination is compulsory. Similarly, women in a study by Pool *et al.* described vaccination as the “law” of the hospital or government [16].

### Potential constraints to child immunization

At the same time, the quality and accessibility of vaccine services at health care facilities surfaced as a significant constraint in the Chókwè and Massinga study. Study participants cited struggles with long queues; travel over long distances; the need to work in the fields in the morning, thus missing clinic hours; and wasted trips to health care facilities when they arrived late.

Community members suggested that a greater use of mobile brigades might alleviate some of the constraints, particularly those related to distance. However, this may lead to understaffed facilities, missed follow-up appointments, and scheduling conflicts with the planting or rainy season. Nonetheless, mobile brigades fill a definite need. Cockcroft *et al.* demonstrated that in Pakistan, district-level vaccination patterns can vary considerably, and that in rural study sites, the presence of a government health facility with vaccination services within 5 kilometres roughly doubled the likelihood that a child would receive the measles vaccine [17].

### Addressing gaps in information

Studies support the notion that targeted communication will be needed to build trust in vaccines among groups that may question them. Reassurances may need to emphasize that the vaccine is licensed and approved by the national government [15,18-20]. Chókwè and Massinga study participants said they would want to know where the vaccine was tested, who would be eligible for vaccination, and why vaccine developers seem to be focussing on children when adults get sick with malaria as well. Participants said they would also have questions about possible vaccine side effects and duration of protection. In Pool’s study, a targeted campaign designed to address concerns, anxieties, and the need for reassurances in the Mozambique intermittent preventive treatment for infants (IPTi) trial contributed to a reversal in the initial rejection of the intervention [16].

### Key messages

Researchers suggest that demand for vaccinations can be enhanced if parents hold positive perceptions of vaccines, trust in health workers, have access to education materials, and have confidence in those who deliver information [21]. Participants in this study cautioned that malaria vaccine messages need to be readily visible or audible at the community level through existing communications channels and in local languages. Participants recommended that messages build on existing community confidence in childhood vaccination and suggested that health information campaigns be carried out by teams of health ministry officials and traditional leaders who go door to door.

Messages would need to address information gaps highlighted in this study. For example, they should address gaps in information about convulsions and other malaria-like symptoms. Many participants in Chókwè and Massinga reported that traditional healers are sought for convulsions, which are often associated with the full moon. Other studies have shown that severe febrile illness accompanied by convulsions or anaemia is frequently not recognized as malaria, and that the child may not be taken promptly to a health facility for treatment but taken to a traditional healer instead [22-24].

Messages would also need to address long-held concerns about vaccine side effects and the perceived risks related to vaccination. Messages would also need to address why vaccines are directed at specific populations, the importance of continuing with malaria prevention and treatment efforts, the characteristics of a particular vaccine, and expectations around vaccine efficacy (see Table 3). Messages around vaccine efficacy or partial efficacy may prove to be challenging, but the findings in this study and two studies related to IPTi provide some evidence of how effective messaging may be crafted. Asked about their understanding of the concept, several participants in the Chókwè and Massinga study spoke of vaccines that worked for a “short time” or “part of the time.” Mothers in Pool’s study saw IPTi as some form of immunization that would make the child more resistant to malaria-related complications or death. The authors in that study found that the idea of partial prevention was strengthened by the recall of information given at enrolment in the trial, the fact that IPTi was being tested, and the advice that existing preventive measures should still be used [16]. Another study on IPTi in five African countries [25] showed that mothers tended to view all prevention practices—traditional or biomedical—as partial, and generally did not think that receiving IPTi meant that their child would no longer get malaria.

### Understanding the primary audience

There is clear evidence of the centrality of mothers to a well-designed health communications strategy. In Chókwè and Massinga, the mother—because of her role as primary caregiver and the person who spends the most time with the children—is primarily responsible for making health-related decisions, including child immunization. Similarly, Cockcroft *et al*. found that children whose mothers reported discussing vaccination within the family were more likely to have received the measles vaccine [17].

### Information channels and trusted sources

Stakeholders in the Chókwè and Massinga study underscored the importance of building on familiar practices and using trusted sources to influence decisions on child vaccination. Religious and other leaders as well as traditional healers were seen as trusted sources of health information in the community. These individuals appeared eager to collaborate with health service providers, to provide accurate information about malaria to the community, and to emphasize the benefits of child immunization through education campaigns. Recognizing and building on the important role played by traditional healers in the care of sick children, especially in rural areas, would be important to the success of a communications strategy for vaccine introduction.

### Study limitations

To meet study objectives, criterion-based sampling techniques were utilized. Due to the small sample size in only two regions of Mozambique, caution should be used when generalizing the results of this study. In addition, although findings in key areas are consistent with other studies reported here, some findings may have the potential for social desirability bias—meaning that in some situations, such as inquiring about a vaccine that does not yet exist, participants may have been inclined to give socially desirable responses rather than describe actual beliefs and practices. That said, community perceptions of child immunization in general and past experiences with child vaccinations were carefully examined to provide a broader understanding of likely acceptance of a new malaria vaccine.

## Conclusions

For a malaria vaccine communications strategy to be effective in Mozambique, its implementation would need to build on existing immunization efforts, familiar practices, and trusted sources for delivering health information, and to involve stakeholders in planning and implementation at both the national and district levels. Participants in this study confirmed that current government dissemination structures for health information are reliable and well-recognized at the community level. A communications strategy should also take into account the influential role played by traditional leaders and traditional healers and involve them as key communicators on issues related to vaccine services as well as malaria prevention and treatment. As such, it would be important to address gaps in information about malaria symptoms and the various causes of convulsions at the community level, including among traditional healers. Translation of information about a future vaccine into local languages and involvement of local leadership in the identification and design of messages would be essential.

## Abbreviations

FGD: Focus group discussion; FHI: Family Health International; IDI: In-depth interview; IPTi: Intermittent preventive treatment for infants; ITN: Insecticide-treated net; MVI: PATH Malaria Vaccine Initiative; TB: Tuberculosis; WHO: World Health Organization.

## Competing interests

The authors hereby declare that they do not have any competing interests.

## Authors’ contributions

AB (principal investigator) was responsible for overall study design and led the writing of the manuscript. FG (co-principal investigator) oversaw all field research activities and participated in analysis and write-up of findings. KL assisted in study design and field researcher trainings, led the analysis, and participated in the write-up of findings. JC oversaw management of the field portion of the study and assisted in analysis and drafting and reviewing the manuscript. YC, AB, and ABN provided technical oversight to the study, including reviewing the protocol, checking data quality, providing input on the analysis plan, and writing and reviewing parts of the manuscript. All authors read and approved the final manuscript.

## References

[B1] World Health Organization (WHO) World Malaria Report 2011 2011 Geneva: WHO

[B2] Ministry of Health (MOH) [Mozambique] Joint Evaluation of Health Sector Performance: Annual Report 2011 2011 Maputo, Mozambique: MOH

[B3] Mabunda SJA The epidemiology and the burden of malaria in Mozambique 2006 Universitat de Barcelona: PhD thesis In Silva JR, Ramos S, Machado M, Moura DF, Neto Z, Canto-Cavalheiro MM, Figueiredo P, Rosario VE, Amaral AC, Lopes D: A review of antimalarial plants used in traditional medicine in communities in Portuguese-speaking countries: Brazil, Mozambique, Cape Verde, Guinea-Bissau, São Tomé and Príncipe and Angola.Mem Inst Oswaldo Cruz [Rio de Janeiro] 2011, 106(Suppl 1):142–158

[B4] Malaria Vaccine Technology Road Maphttp://www.malariavaccine.org/malvac-roadmap.php

[B5] Malaria Vaccine Decision-Making Frameworkhttp://malvacdecision.net/

[B6] World Health Organization Regional Office for Africa (WHO AFRO) Africa WROf: The Work of WHO in the Africa Region, 2008–2009 2010 Republic of Congo: WHO AFRO, Brazzaville

[B7] World Health Organization (WHO) Department of Immunization, Vaccines and Biologicals Adding a Vaccine to a National Immunization Programme: Decision and Implementation 2005 Geneva: WHO

[B8] Bingham A Drake JK LaMontagne DS Sociocultural issues in the introduction of human papillomavirus vaccine in low-resource settings Arch Pediatr Adolesc Med 2009 163 5 455 461 19414692 10.1001/archpediatrics.2009.50

[B9] Green LW Kreuter MW Health Program Planning: An Educational and Ecological Approach 2005 4 New York: McGraw-Hill Companies

[B10] Kane MA Sherris J Coursaget P Aguado T Cutts F HPV vaccine use in the developing world Vaccine 2006 24 Suppl 3 132 139 10.1016/j.vaccine.2006.05.128 16950000

[B11] Zimet GD Liddon N Rosenthal SL Lazcano-Ponce E Allen B Psychosocial aspects of vaccine acceptability Vaccine 2006 24 Suppl 3 201 209 10.1016/j.vaccine.2006.06.017 16950008

[B12] Dodson B Simelane H Tevera D Green T Chikanda A de Vletter F Gender Migration and Remittances in Southern Africa 2008 Cape Town South Africa: Southern African Migration Project

[B13] Lewis MP Languages of Mozambique Ethnologue: Languages of the World 2009 16 Dallas, TX: SIL International

[B14] Nichter M Vaccinations in the Third World: a consideration of community demand Soc Sci Med 1995 41 617 632 7502096 10.1016/0277-9536(95)00034-5

[B15] Katahoire RA Jitta J Kivumbi G Murokora D Arube WJ Siu G Arinaitwe L Bingham A Mugisha E Tsu V LaMontagne DS An assessment of the readiness for introduction of the HPV vaccine in Uganda Afr J Reprod Health 2008 12 159 172 19435020

[B16] Pool R Muguambe K Macete E Aide P Juma G Alonso P Mendendez C Community response to intermittent preventive treatment delivered to infants (IPTi) through the EPI system in Manhiça. Mozambique Trop Med Int Health 2006 11 1670 1678 17054746 10.1111/j.1365-3156.2006.01725.x

[B17] Cockcroft A Andersson N Omer K Ansari NM Khan A Chaudhry UU Ansari U One size does not fit all: local determinants of measles vaccination in four districts of Pakistan BMC Int Health Hum Rights 2009 9 Suppl 1 S4 19828062 10.1186/1472-698X-9-S1-S4 PMC3226236

[B18] Nguyen QN LaMontagne DS Bingham A Rafiq M Le TPM Nguyen TPL Nguyen CK Duong TH Dang TTH Nguyen TTT Nguyen TH HPV vaccine introduction in Vietnam: formative research findings Sex Health 2010 7 262 270 20719213 10.1071/SH09123

[B19] Jacob M Mawar N Menezes L Kaipilyawar S Gandhi S Khan I Patki M Bingham A LaMontagne DS Bagul R Katendra T Karandikar N Madge V Chaudhry K Paranjape R Nayyar A Assessing the environment for introduction of human papillomavirus vaccine in India Open Vaccine J 2010 3 96 107

[B20] Bartolini RM Drake JK Creed-Kanashiro HM Díaz Otoya MM Mosqueira Lovón NR Penny ME Winkler JL LaMontagne DS Bingham A Formative research to shape HPV vaccine introduction strategies in Perualud Pública Méx 2010 52 226 233 10.1590/s0036-36342010000300007 20485886

[B21] Streefland PH Chowdhury AM Ramos-Jimenez P Quality of vaccination services and social demand for vaccinations in Africa and Asia Bull World Health Organ 1999 77 722 730 10534895 PMC2557734

[B22] Comoro C Nsimba SE Warsame M Tomson G Local understanding, perceptions and reported practices of mothers/guardians and health workers on childhood malaria in a Tanzanian district—implications for malaria control Acta Trop 2003 87 305 313 12875923 10.1016/s0001-706x(03)00113-x

[B23] Pilkington H Mayombo J Aubouy N Deloron P Malaria, from natural to supernatural: a qualitative study of mothers’ reactions to fever (Dienga, Gabon) J Epidemiol Community Health 2004 58 826 830 15365107 10.1136/jech.2003.016089 PMC1763324

[B24] Yé Y Traoré C Meissner P Coulibaly B Becher H Müller O Ability of mothers to diagnose fever and anaemia in their young children, in a malaria-endemic region of West Africa Ann Trop Med Parasitol 2007 101 297 303 17524244 10.1179/136485907X176391

[B25] Gysels M Pell C Mathanga DP Adongo P Odhiambo F Gosling R Akweongo P Mwangi R Okello G Mangesho P Slutsker L Kremsner PG Grobusch MP Hamel MJ Newman RD Pool R Community response to intermittent preventive treatment of malaria in infants (IPTi) delivered through the expanded programme of immunization in five African settings Malar J 2009 8 191 19664250 10.1186/1475-2875-8-191 PMC2734860

